# Effectiveness and Safety of the Intermittently Scanned Continuous Glucose Monitoring System FreeStyle Libre 2 in Patients with Type 2 Diabetes Treated with Basal Insulin or Oral Antidiabetic Drugs: An Observational, Retrospective Real-World Study

**DOI:** 10.3390/jcm13030642

**Published:** 2024-01-23

**Authors:** Matteo Conti, Giulia Massari, Elena Meneghini, Bernadetta Pasquino, Barbara Agosti, Federica Chinotti, Basilio Pintaudi, Angela Girelli, Federico Bertuzzi

**Affiliations:** 1 Diabetes Unit, Niguarda Cà Granda Hospital, 20162 Milan, Italy; m.conti69@campus.unimib.it (M.C.); elena.meneghini@ospedaleniguarda.it (E.M.); basilio.pintaudi@ospedaleniguarda.it (B.P.); 2 Department of Medicine and Surgery, University of Milan-Bicocca, 20126 Milan, Italy; 3 Diabetes Unit, Spedali Civili di Brescia, 25123 Brescia, Italy; giulia.massari.pq4o@rg.omceo.it (G.M.); bernadetta.pasquino@asst-spedalicivili.it (B.P.); barbara.agosti@asst-franciacorta.it (B.A.); angela.girelli@gmail.com (A.G.); 4 University of Milan, 20122 Milan, Italy; federica.chinotti@unimi.it

**Keywords:** flash glucose monitoring, basal insulin, type 2 diabetes mellitus

## Abstract

Intermittently Scanned Continuous Glucose Monitoring (isCGM) devices are increasingly being used in patients with type 2 diabetes mellitus (T2DM) on insulin therapy for their benefits regarding disease management. Evidence of isCGM use in patients with T2DM on basal or non-insulin therapy is lacking. This study aimed at assessing the efficacy and safety of isCGM in this population. This was an observational, retrospective, real-world study enrolling patients with T2DM who were starting the use of isCGM. Data from medical records (i.e., demographics, clinical characteristics, laboratory assessments, and isCGM metrics) were collected over three time periods (baseline, 3 and 6 months). The endpoints were glycated haemoglobin (HbA1c) changes and changes in isCGM metrics as defined by the International Consensus from baseline to 3 months and 6 months. Overall, 132 patients were included (69.5% male; mean age 68.2 ± 11.0 years; mean disease duration 19.0 ± 9.4 years; 79.7% on basal insulin ±non-insulin therapy; mean baseline HbA1c 8.1% ± 1.3%). The estimated mean change in HbA1c was statistically significant at three (−0.4 ± 1.0%; *p* = 0.003) and six months (−0.6 ± 1.3%; *p* < 0.0001). In conclusion, isCGM proved to be effective and safe in improving glycaemic control in patients with T2DM on basal insulin or non-insulin therapy.

## 1. Introduction

Intermittently Scanned Continuous Glucose Monitoring (isCGM), also known as Flash Glucose Monitoring (FGM), is a well-known valuable tool for managing patients with diabetes undergoing insulin treatment, including those with Type 2 Diabetes Mellitus (T2DM).

Intermittently Scanned Continuous Glucose Monitoring devices provide continuous and real-time glucose monitoring, allowing individuals to track their glucose levels throughout the day and night without the need for finger pricking [1]. This can offer a more comprehensive view of blood sugar patterns as compared to traditional fingerstick testing, and no calibration is required. Real-time Continuous Glucose Monitoring (rt-CGM) does not require manual scanning, and it provides predictive alarms for hypo- or hyperglycaemia, thus resulting in being superior to isCGM in terms of glycaemic control and cost-effectiveness [2]. Indeed, isCGM devices are cheaper and often easier to use and to apply, which makes them a viable alternative to rt-CGM devices in diabetes management. The efficacy of both isCGM and rt-CGM in type 1 diabetes mellitus has been confirmed by a large body of evidence [1,3]. 

Data regarding type 2 diabetes mellitus are less complete as compared to those regarding type 1 diabetes mellitus. In adults with type 2 diabetes under multiple daily insulin injections (MDI), isCGM has been observed to improve glycated haemoglobin (HbA1c) [4], with reductions in hypoglycaemia [5,6] and increased treatment satisfaction [4,5]. Therefore, the recent American Diabetes Association (ADA) guidelines have suggested that isCGM should be offered for diabetes management in patients with type 2 diabetes on multiple daily injections or continuous subcutaneous insulin infusion who are capable of using the devices safely [7]. However, evidence regarding the use of isCGM in patients under basal insulin or noninsulin therapy is more limited [8,9,10,11,12,13,14,15,16,17]. Initial evidence, both from randomized controlled clinical trials and real-world studies, showed that isCGM use was mainly associated with glycated haemoglobin improvement [8,9,10,11,12,13,14,15,16], a reduction of acute diabetes-related events (ADEs; i.e., severe hypoglycemia and diabetic ketoacidosis) [10] and a reduction of the rate of hospitalization for ADEs [9,10,12]. However, the data are not homogenous, and the majority of the studies are on national databases [8,9,10,11,12]. Therefore, the real efficacy of isCGM in patients under basal insulin or noninsulin therapy has yet to be proven. 

In this study, we aimed at assessing the efficacy and safety of isCGM Freestyle Libre 2 in a population of patients treated with non-insulin therapy or basal insulin plus non-insulin therapy.

## 2. Materials and Methods

This was an observational, retrospective, real-world study enrolling patients with T2DM followed by the Niguarda Ca’ Granda Hospital, Milan, Italy and Brescia Hospital, Italy. A consecutive sample of patients with T2DM who were starting with the use of Freestyle Libre 2 was included in the study. Only patients who had received diabetological continuous assistance for at least one year were included in the study. The exclusion criteria were T2DM treated with multiple daily injections, type 1 diabetes, gestational diabetes, and other types of diabetes. All participants, as a standard procedure at each visit, were advised to follow lifestyle modifications according to standard care. Clinical data at baseline and after three and six months were recorded. Specifically, information regarding the following parameters were collected: age, gender, diabetes duration, height, weight, smoking habits, glycated haemoglobin, fasting plasma glucose, blood pressure, lipid profile (total cholesterol, LDL-cholesterol, HDL-cholesterol, triglycerides), pharmacologic treatments, and diabetes-related complications, such as retinopathy, nephropathy, neuropathy, and macroangiopathy. In particular, macroangiopathy was defined as a history of a cardiovascular event and/or ischemic electrocardiogram abnormalities at rest or during a stress test, the presence of plaques detected by ultrasonographic examination of the carotid arteries or the peripheral arterial vessels, or as the presence of an intima media of thickness >1.5 mm. Neuropathy was diagnosed using the vibration perception test, the monofilament pressure sensation test, or electromyography. Nephropathy was defined as a reduced glomerular filtration rate (<60 mL/min) or an increased urinary albumin excretion (albuminuria) diagnosed either if the urinary albumin concentration was >30 mg/L, the urinary albumin excretion rate was >20 μg/min, or the urinary albumin-to-creatinine ratio was >2.5 mg/mmol in men and 3.5 mg/mmol in women. Retinopathy was detected using high-quality fundus photographs.

The study endpoints were to evaluate changes in HbA1c levels and changes in CGM metrics as defined by the International Consensus on Time in Range [18] from baseline to a 3- and a 6-month follow-up. In particular, % time in range (%TIR) (70–180 mg/dL), % time above range 1 (%TAR1) (180–250 mg/dL), % TAR2 (>250 mg/dL), % time below range 1 (%TBR1) (55–70 mg/dL) and % TBR2 (<55 mg/dL) were assessed at a 3- and a 6-month follow-up.

The possible occurrence of side effects related to the use of the isCGM system was investigated at each visit. All the clinical data collected in the study were analyzed anonymously. The local Ethics Committees approved the protocol, and all participating patients gave written informed consent. The study was carried out according to the Helsinki Declaration. 

### Statistical Analyses

Data were expressed as means ± standard deviation for the continuous variables and percentages for the categorical variables. The Kolmogorov–Smirnov test was used to test the normality of distribution of the continuous variables. Clinical and demographic characteristics were compared using the Wilcoxon signed-rank test. As this was a feasibility study, no prior power calculation was carried out. A *p* value < 0.05 was considered to be statistically significant. The analyses were carried out using SPSS version 21.0 (SPSS, Inc., Chicago, IL, USA).

## 3. Results

Overall, 132 patients were enrolled in the study; 79.7% of them were being treated with basal insulin plus non-insulin therapy and only 20.3% with non-insulin therapy. Ninety-one (69.5%) medical records were for male individuals. The mean age at the start of the device use was 68.2 ± 11.0 years, with a mean disease duration of 19.0 ± 9.4 years and a baseline HbA1c of 8.1 ± 1.3%. The most frequent comorbidity was hypertension (65.9%), and more than one third of the population presented with a history of ischemic heart disease at baseline (35.5%). The baseline characteristics of the patients and their comorbidities are reported in Table 1.

After the introduction of isCGM, the estimated mean change in HbA1c from baseline was statistically significant at three (−0.4 ± 1.0%; *p* = 0.003) and at six months (−0.6 ± 1.3%; *p* < 0.0001). The HbA1c level significantly decreased at the 3- (7.51 ± 0.91%, *p* = 0.003) and at the 6-month follow-ups (7.54 ± 0.96%, *p* < 0.001) (Table 2).

Similarly, considering only patients being treated with basal insulin therapy plus non-insulin, after the introduction of isCGM, the HbA1c level significantly decreased at the 3- (7.99 ± 1.99% vs. 7.54 ± 0.91%, *p* = 0.015) and the 6-month follow-ups (7.70 ± 0.96%, *p* = 0.001 at 6 months).

In Table 3, the different therapies at baseline and at the 3- and the 6-month follow-ups were reported. They did not show a consistent modification during the follow-up. The significant mean difference in HbA1c levels between the baseline and the follow-up visits was not affected by changes in glucose-lowering therapy. In fact, at 6 months, HbA1c significantly decreased compared to baseline, both in the patient group with therapy modification (7.58 ± 1,00 vs. 7.95 ± 1.30, *p* = 0.01) and in the group without therapy modification (7.52 ± 0.94 vs. 7.96 ± 1.33, *p* = 0.006). 

No statistical differences were found in the mean change of TIR, TAR1 and TAR2. Indeed, TBR1 and TBR2 significantly decreased at 6 months compared to 3 months values (see Table 4). Comparisons were feasible only between the 3- and the 6-month follow-ups, as the CGM metrics at baseline were lacking inasmuch as, at enrollment, patients were not wearing a glycaemic sensor. 

The table shows the time in range (%TIR) (70–180 mg/dL), % time above range 1 (%TAR1) (180–250 mg/dL), % TAR2 (>250 mg/dL), % time below range 1 (%TBR1) (55–70 mg/dL) and % TBR2 (<55 mg/dL). CGM metrics were assessed at a 3- and a 6-month follow-up.

No side effects related to the use of isCGM were reported. There were no significant changes in the lipid profile, prevalence of comorbidities and chronic diabetes complications, or in the hypoglycaemic rate. No episode of severe hypoglycemia occurred throughout the follow-up period.

## 4. Discussion

Evidence supporting the use of CGM in T2DM patients is increasing; the overall positive results have led to considering the possible economic implications resulting from the large number of T2DM patients who might benefit from these devices [2]. Intermittently scanned-CGM is a feasible alternative to rt-CGM, being more convenient, easier to apply and use, thinner and having reduced dimensions. 

However, scientific evidence regarding the use of isCGM in type 2 diabetes mellitus is more limited. Controlling blood glucose levels in patients affected by T2DM using basal insulin therapy and no rapid insulin can be challenging [19]. Although many patients with T2DM routinely carry out blood glucose monitoring (BGM) in the morning, postprandial monitoring is often not performed, leading to postprandial glycemia being underestimated. In addition, the assessment of postprandial glycaemia might be delayed and not useful for showing the glycaemic peaks. Furthermore, while basal insulin addresses fasting blood sugar levels, it does not account for the postprandial glucose spikes that occur after eating. Administering too much basal insulin or not adjusting the dose properly can increase the risk of hypoglycaemia without reducing postprandial glucose excursions. Patients with T2DM may require additional oral medications to address these spikes effectively. Alternatively, they can modify the meal composition or reduce the amount of food [20]. 

The use of isCGM in this set of patients can improve the management of their glycaemic control as it facilitates the basal insulin titration necessary for controlling the fasting glycaemic concentration, it alerts the patients to postprandial glycaemic changes, and it enables them to personalize nutrition therapy based on individual glycaemic patterns in order to reduce glycaemic oscillations. The role of isCGM in motivating eating behavior modifications has already been described in the literature. It has been shown that isCGM, together with the Self-Evaluation Of Unhealthy foods by Looking at postprandial glucose (SEOUL) algorithm, was able to improve HbA1c values [15]. According to this algorithm, the routine use of isCGM led patients to qualitative and quantitative modifications in their eating behavior, thus reducing postprandial glycaemic oscillations. Polonsky and colleagues integrated isCGM into diabetes self-management education and support (DSMES) programs, and achieved a similar improvement in glycaemic control. There was a significant gain in % TIR and a parallel drop in % TAR, together with an overall increase in well-being, and an improvement in healthy eating [21]. This conclusion supported a new approach to DSMES, a method that integrates isCGM with a highly interactive and engaging patient-driven “discovery learning” approach to education. A prerequisite for the success of this strategy is a good level of acceptance of the patients toward this device; patients affected by T2DM are among those patients reporting higher “convenient” and “discreteness” scores regarding isCGM [22]. Therefore, isCGM appeared to play an important educational role, allowing patients to have a better understanding of their glucose levels throughout the day, helping them make informed decisions regarding their diet, physical activity, medication adjustments, and lifestyle changes, alerting patients to impending hypoglycaemia and hyperglycemia, and encouraging active participation in diabetes self-management. Thus, patients are more likely to take ownership of their condition, monitor their glucose levels, and adhere to treatment plans.

In the present paper, the authors showed that the use of isCGM in T2DM patients on basal insulin or only on oral drugs improved overall glycaemic control. The effect was independent of eventual therapy modifications.

Specifically, the reduction in HbA1c after 3 months of isCGM use was about 0.4% in our study, in range with several scientific evidences, such as the works of Eeg-Olofsson et al. and Wada et al. [11,17]. In the literature, data from other cohorts show greater reductions in glycated haemoglobin during isCGM use across a similar follow-up period [13,14]. This difference might be explained by the higher mean HbA1c levels of the population at baseline in these studies, as higher baseline values are reported to be associated with significantly larger reductions [4]. Indeed, the decrease in HbA1c achieved in our population can be defined as being significant, considering the non-severe impairment of glycemic compensation at baseline. According to a systematic review and meta-analysis of isCGM use in type 1 and type 2 diabetes, the use of these devices results in a 0.4% reduction in HbA1c for each 1% increase in baseline levels over 7.2% [22]. The results of the present study can be considered in line with this meta-analysis, as the mean glycated haemoglobin was around 8.1% in our cohort at baseline. In addition, a large number of patients in the present cohort can be considered fragile (mean age: 68.2 ± 11 years; heart disease at baseline: 35.5%; arteriopathy at baseline: 27.8%; nephropathy at baseline: 24.0%) with a less ambitious HbA1c target according to the ADA Standard.

Finally, the efficacy in reducing HbA1c values was accompanied by a good safety profile, evidenced by the absence of episodes of severe hypoglycaemia in the study population throughout the follow-up period and by the absence of differences in TBR1 and TBR2 parameters at 3 and 6 months of observation.

## 5. Conclusions

In conclusion, this study confirmed that the use of isCGM could, due to its effectiveness and safety, be considered a useful tool for improving overall glycaemic control in patients with type 2 diabetes who are being treated with non-insulin or basal insulin therapy.

However, this study has some limitations. The most important one is that the study used a single-arm retrospective chart review methodology, which, by definition, precluded a SMBG control group as a comparison for the effectiveness of the intervention. The authors did not evaluate or record lifestyle changes in the patients enrolled, and the research period was only 6 months, making it unclear whether the improvement in glycaemic control using isCGM would last longer. However, data from patients where a 12-month follow-up is available seem to confirm the trend of glycated hemoglobin reduction shown in this study, although the numerosity does not yet allow for an appropriate statistical analysis. 

These limitations highlight the need for other studies to further address this topic.

## Figures and Tables

**Table 1:** Patients’ baseline characteristics (a) and comorbidities (b).

a. Patients’ Characteristics	Mean ± SD
Age	68.2 ± 11.0
Duration of diabetes (yrs)	19.0 ± 9.4
Weight (kg)	79.5 ± 19.5
Height (cm)	168.7 ± 8.5
Systolic blood pressure (mmHg)	136 ± 18
Diastolic blood pressure (mmHg)	75 ± 9
Albuminuria (mg/L)	140.4 ± 456.2
Glycated haemoglobin (%)	8.1 ± 1.3
Fasting plasma glucose (mg/dL)	154.8 ± 44.4
Total cholesterol (mg/dL)	153.6 ± 42.0
LDL (mg/dL)	81.4 ± 38.0
HDL (mg/dL)	46.9 ± 14.8
Triglycerides (mg/dL)	139.2 ± 74.4
**b. Patients’ comorbidities**	%
Ischemic cardiopathy	35.5
Hypertension	65.9
Arteriopathy	27.8
Nephropathy	24.0
Retinopathy	16.8
Neuropathy	11.2
Severe hypoglycaemia	4.9

**Table 2:** HbA1c values after isCGM use at different follow-ups.

	3 Months	6 Months
Total	7.51 ± 0.91 (0.003)	7.54 ± 0.96 (0.001)
Basal insulin + Oral hypoglycemic drugs	7.54 ± 0.91 (0.01)	7.70 ± 0.96 (<0.001)
Only Oral hypoglycemic drugs	7.37 ± 0.91 (0.003)	7.13 ± 1.06 (0.30)

Values are median ± standard deviation, and data are in %. In parentheses: *p* values of comparison vs. basal HbA1c.

**Table 3:** Patient therapy during follow-up.

	Baseline (%)	3 Months (%)	6 Months (%)
Basal insulin	79.7	86.3	78.8
Metformin	62.4	52.8	61.0
Pioglitazone	4.9	6.0	5.1
Sulfonylureas	17.9	9.8	16.3
Acarbose	4.1	2.0	10.1
DPP4 inhibitors	17.9	23.5	18.8
SGLT2 inhibitors	33.3	41.2	40.0
GLP-1 analogues	50.8	57.7	54.3

**Table 4:** CGM metrics at 3- and 6-month follow-ups.

	3 Months (%)	6 Months (%)	*p*
TIR	62.3	61.6	0.28
TAR1	27.4	27.8	0.22
TAR2	7.1	8.3	0.57
TBR1	2.2	1.9	0.001
TBR2	1.1	0.4	0.001

## Data Availability

The datasets analyzed in the current study are available from the corresponding author on reasonable request.
